# Dengue diagnosis and impact on clinical management: A literature review

**DOI:** 10.1371/journal.pntd.0013196

**Published:** 2025-06-30

**Authors:** Solenne Robert, Potiandi Serge Diagbouga, Arthur Diakourga Djibougou, Danielle Guy, Robert Bagnall, Fanette Ravel

**Affiliations:** 1 Global Medical Affairs, bioMérieux SA, Marcy l’Etoile, France; 2 Institut de Recherche en Sciences de la Santé (IRSS), Centre National de la Recherche Scientifique et Technologique (CNRST), Ouagadougou, Burkina Faso; 3 Health Economics and Market Access, Amaris Consulting, Barcelona, Spain; University of Khartoum, SUDAN

## Abstract

**Background:**

Dengue is a re-emerging infectious disease that poses substantial challenges to healthcare systems in endemic regions, such as West Africa. Owing to its nonspecific and overlapping clinical symptoms – including fever, rash, headache, joint pains, nausea, vomiting – many cases go unrecognized or are misdiagnosed. Consequently, patients are often inappropriately treated with antimalarial or antibiotic therapies. Such mismanagement not only affects patient outcomes but also contributes to the development of antimicrobial and antimalarial drug resistance within these populations. This literature review aimed to describe the patterns and impact of dengue diagnosis in West Africa.

**Methods:**

A comprehensive electronic database search of MEDLINE and Embase was conducted using keywords related to dengue, chikungunya, acute febrile illness, diagnostic strategies, and clinical management. Additional manual searches was performed through Google Scholar and relevant conference proceedings. Eligible studies included observational, real-world evidence, or interventional research conducted in West Africa involving adult patients diagnosed with dengue or chikungunya. Inclusion criteria required studies to report on diagnostic approaches and/or clinical management strategies. Due to the limited availability of data on chikungunya, this review focused exclusively on dengue.

**Results:**

Ten studies from Burkina Faso, Ivory Coast, Senegal, and Nigeria were included in this review. Rapid serologic testing (DENGUE NS1, IgG, IgM) was the most frequently used diagnostic tools, used in 60% of the studies. Clinical management of dengue primarily involved the administration of antipyretics, fluid therapy, and blood transfusions when necessary. In five studies, antimalarial treatments were systematically prescribed despite negative malaria results. Additionally, two studies reported the use of antibiotics without confirmed bacterial infection or supporting biological diagnosis, indicating inappropriate antibiotic use.

**Conclusions:**

Evidence on dengue diagnosis and related clinical management in West Africa remains limited. The available data indicate a widespread underuse of diagnosis tools and frequent misuse of antimalarial and antibiotic therapies in the management of dengue. Future studies should prioritize evaluating the impact of accurate differential diagnosis between dengue and malaria on patient care, particularly regarding the inappropriate use of antibiotics and antimalarials. Moreover, integrating routine diagnostic testing into standard clinical practice, as recommended by health authorities, could significantly improve current management. This would enable clinicians to more accurately diagnose dengue, malaria, other febrile illnesses, and potential co-infections, ultimately reducing the misuse of antimicrobial treatments.

## Author summary

Dengue is a mosquito-borne viral disease that causes symptoms such as fever, headache, rash, joint pain, and nausea. These symptoms are very similar to those of other common illnesses like malaria, making it difficult to diagnose correctly. In West Africa, many dengue cases go unrecognized or are confused with malaria or bacterial infections. A review of studies from Burkina Faso, Ivory Coast, Senegal, and Nigeria found that healthcare providers often rely on symptoms alone rather than using proper tests to confirm dengue. In many cases, patients were given antimalarial or antibiotic treatments even when tests for malaria were negative or there was no sign of bacterial infection. This inappropriate use of medication can harm patients and contribute to drug resistance. The most commonly used test in the reviewed studies was a rapid blood test for dengue. However, these tests were not used in all healthcare settings. The review highlights the urgent need to improve diagnostic practices in West Africa. By using proper testing, doctors can better distinguish dengue from other diseases. This would lead to more accurate treatment, reduce the misuse of antibiotics and antimalarials, and improve health outcomes in the region.

## Introduction

Arboviral diseases are caused by a diverse group of viruses transmitted to humans through the bite of infected arthropods, primarily mosquitoes or ticks. In recent years, the incidence and burden of arboviral have increased substantially, presenting a growing global public health challenge [1]. Dengue virus (DENV), a member of the *Flaviviridae* family, is one of the most prevalent arboviruses and exists in four serotypes (DEN 1, DEN 2, DEN 3, and DEN 4) [2,3]. It is primarily transmitted to humans through the bites of infected *Aedes* mosquitoes (*Ae. aegypti* or *Ae. albopictus)*. According to the World Health Organization (WHO), an estimated 390 million dengue virus infections occur annually (95% confidence interval: 284–528 million), with approximately 96 million (67–136 million) resulting in clinical manifestations of varying severity [4,5]. Reported attack rates for dengue have exceeded 80% in certain outbreaks [6,7]. Over 7.6 million dengue cases were reported to WHO in 2024, including 3.4 million confirmed cases, over 16 000 severe cases, and over 3000 deaths [8]. Despite this high burden, co-infections involving arboviruses and malaria parasites are rarely documented in the scientific literature, likely due to under-reporting and the limited availability of diagnostic infrastructure for arboviral infections in Sub-Saharan Africa [9]. Additionally, factors such as a population growth, rapid and unregulated urbanization, human mobility, and climate change have contributed to increased arbovirus transmission by enhancing vector survival and extending transmission seasons [10,11]. The clinical overlap with other febrile illnesses, particularly malaria, combined with limited diagnostic access and inadequate surveillance systems, suggests that the true burden and socio-economic impact of dengue in this region are significantly underestimated [12].

Dengue is endemic in West Africa, a region experiencing the fastest population growth globally. This rapid demographic expansion, coupled with unregulated urbanization, poses significant challenges to effective dengue surveillance and vector control efforts – both of which are critical for mitigating transmission risk [11]. Historically, the intense focus on malaria as the predominant cause of febrile illness, combined with limited healthcare resources, has contributed to the neglect of alternative etiologies such as dengue [13,14]. Consequently, many febrile illnesses are presumptively diagnosed and managed as malaria, largely due to the high endemicity of malaria in sub-Saharan Africa and earlier WHO recommendations favoring presumptive antimalarial treatment [15]. This diagnostic bias often results in dengue cases going unrecognized, as clinical features such as fever, myalgia, and rash overlap with those of malaria. For instance, a study conducted at a primary health center in Lagos, Nigeria, found that 83% of febrile children received artemisinin-based combination therapies despite having negative malaria test results [16].

Suboptimal clinical management of febrile illnesses – particularly the empirical use of antimalarial drugs without laboratory confirmation – contribute to the development of drug resistance [17,18]. In West Africa, febrile patients frequently treated with inappropriate antimalarial therapy in the absence of diagnostic testing, often in combination with unnecessary antibiotic prescriptions [19]. The misuse of antibiotics is a major driver of antimicrobial resistance (AMR), which occurs when the effectiveness of antimicrobial agents diminishes due to overuse and misuse. AMR represents one of the most pressing global health threats of the 21st century, with Africa experiencing the highest mortality rates attributed to AMR worldwide [20,21].

To improve the clinical management of febrile illness, it is essential to implement robust adequate diagnostic policies. In the case of dengue, NS1 antigen and IgM antibody tests are valuable for early diagnosis, while IgG antibody tests help identify previous infections [22,23]. This distinction is crucial, as secondary dengue infections are associated with a higher risk of severe disease. In endemic regions, individuals are often sequentially exposed to multiple dengue virus serotypes, increasing their susceptibility to severe clinical outcomes [24].

The lack of specific antiviral therapies for dengue infection presents further challenges to effective clinical management. Supportive care, including the use of analgesics and antipyretics, remains the cornerstone of treatment and is frequently self-administered or prescribed by healthcare workers in addition to judicious hydration to prevent shock or organ damage [25]. However, the antiplatelet properties of acetylsalicylic acid and other nonsteroidal anti-inflammatories drugs pose significant risks for patients with dengue, as they can exacerbate bleeding tendencies and potentially lead to fatal hemorrhagic complications that could induce severe dengue and potentially result in fatal hemorrhage [4]. Consequently, accurate differential diagnosis is essential for guiding appropriate treatment and preventing severe outcomes.

While several studies have assessed the role of differential malaria diagnosis in improving the clinical management of febrile illnesses [26], no study has been conducted on the impact of dengue diagnosis on patient clinical management. This review aims to address this gap by examining the impact of dengue diagnosis on clinical and patient management in West Africa, with a specific focus on diagnostic practices and the misuse of antimalarials and antibiotics.

## Methods

This comprehensive literature review was conducted as a literature review and analysis, and was reported in accordance with the Preferred Reporting Items for Systematic Reviews and Meta-Analyses (PRISMA) guidelines.

### Eligibility criteria

Eligible publications included observational or interventional studies (e.g., cross-sectional studies and retrospective analysis) conducted in West Africa, involving patients presenting with suspected clinical signs consistent with dengue infection. To be included, studies were required to report on clinical management strategies alone or in conjunction with diagnostic methods. Seroprevalence studies that exclusively reported diagnostic methods without addressing clinical management were excluded, as they did not provide information on clinical management.

### Search strategy

An electronic database search of MEDLINE and Embase was performed in Embase on August 18, 2022. The search strategy employed a combination of keywords related to dengue, chikungunya, febrile illness, diagnostic strategies, and clinical management (**Table 1**) to identify relevant publications.

**Table 1:** Embase search strategy.

PICOS Element	#	Embase search syntax*	Number of hits
Population	1	‘arbovirus’/exp OR ‘febrile illness’ OR ‘dengue shock syndrome’/exp OR ‘dengue’/exp OR ‘dengue hemorrhagic fever’/exp OR ‘dengue virus’/exp OR ‘dhf’:ti,ab,kw OR ‘dss’:ti,ab,kw OR ‘chikungunya’/exp OR ‘chikungunya fever’/exp OR ‘chikungunya virus’/exp OR (‘coinfection’ NEAR/2 (dengue OR chikungunya))	71,670
Interventions	2	diagnos* OR ‘rapid diagnostic test’:ti,ab,kw OR ‘rapid test’:ti,ab,kw OR ‘diagnostic assay’:ti,ab,kw OR (test NEAR/2 serolog*) OR ‘diagnostics’/exp OR ‘antibody test’ OR ‘antigen test’ OR ns1 OR igg OR igm OR ‘dengue antigen’ OR ‘anti-dengue’ OR ‘chikungunya antigen’ OR ‘anti-chikungunya’	11,031,516
Outcomes	3	differential diagnosis’/exp OR ‘clinical management’/exp OR ‘disease management’/exp OR (‘antibiotic’ NEAR/2 (use OR misuse OR prescription OR treatment OR therapy)) OR (‘antimalarial’ NEAR/2 (use OR misuse OR prescription OR treatment OR therapy)) OR ‘case management’/exp OR ‘hospitali*ation$’ OR ‘heathcare resource use’ OR ‘hcru’ OR ‘impact’:ti,ab,kw OR ‘treatment’:ti,ab,kw OR ‘co-infection:ti,ab,kw’ OR ‘antimicrobrial resistance’:ti,ab,kw OR ‘misdiagnos*’:ti,ab,kw OR ‘missclassification’:ti,ab,kw OR ‘impact evaluation’:ti,ab,kw	10,941,532
Geography	4	‘West Africa’:ti,ab OR ‘sub-Saharan Africa’ OR (‘Ivory Coast’ OR ‘Burkina Faso’):ti,ab,ad,ff	52,985
Final limit to articles	5	#1 AND #2 AND #3 AND #4	162

Supplementary searches were also conducted to capture additional literature, including proceedings from the International Conference on Emerging Infectious Diseases and the International Congress on Infectious Diseases, gray literature, and Google Scholar [13,27]. Experts in the field, especially Pr. Sondo, were also contacted to identify potentially relevant unpublished or overlooked studies.

Article screening was performed by one reviewer, with quality checks conducted by a second reviewer. Duplicate records were removed systematically. Any disagreements during the screening or inclusion process were resolved through discussion or, when necessary, by consulting a third reviewer.

### Data analysis

The findings of the review were analyzed descriptively. Data synthesis focused on diagnostic methods, clinical management strategies—including self-medication practices—and the influence of differential diagnosis on treatment decisions.

## Results

### Study characteristics

**Fig 1:**
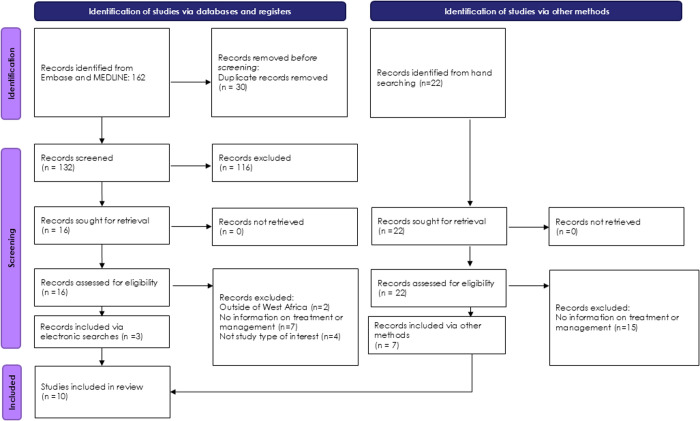
PRISMA diagram.

The study selection process, along with reasons for exclusions, is presented in **Fig 1**, in accordance with the PRISMA 2020 statement [28]. The characteristics of the included studies are summarized in **Table 2**. The electronic database search yielded a total of 132 studies after the removal of duplicates. An additional 22 studies were identified through supplementary searches.

**Table 2:** Characteristics of included studies.

Author, year	Country	Study type	Population and sample size	Conclusions
Zongo, 2018 [31]	Burkina Faso	Cross-sectional	CSPS health professionals (N = 32)	Health professionals were appreciative of dengue RDTs to improve their ability to diagnose and manage dengue. Clinical management of dengue was based on patient symptomology (e.g., analgesics for pain, antipyretics for fever, antibiotics for suspected bacterial infections, or anti-emetics for vomiting). Patients were advised not to self-medicate. Antimalarial drugs were prescribed in suspected false-negative malaria cases.
Sondo, 2017 [30]	Burkina Faso	Cross-sectional	Individuals with confirmed primary dengue diagnosis (positive AgNS1 or IgM; N = 811)	Results indicate that many dengue cases are still treated as malaria even if their malaria tests are negative.
Ridde, 2016 [28]	Burkina Faso	Cross-sectional	Individuals with fever or history of fever within previous week and negative malaria RDT (N = 379)	Despite positive malaria diagnoses, many febrile episodes cannot be attributed to malaria. Nearly one third of patients with a positive dengue diagnosis chose to self-medicate.
Sawadogo, 2016 [29]	Burkina Faso	Cross-sectional	Patients with positive dengue tests (N = 6,200)	PCs played a major role in the management of dengue in hospitalized patients, especially during the outbreak period. Although, in many cases the prescription of PCs was not justified given the shortage of blood supply.
Diallo, 2017 [26]	Burkina Faso	Retrospective analysis	Hospitalized patients with fever and positive AgNS1 (N = 98)	Nearly all patients received rehydration. However, antimalarials and antibiotics were also administered to patients without confirmed malaria diagnosis. Most patients (66%) received antimalarials despite only 43% of patients having it.
Aoussi, 2014 [25]	Ivory Coast	Retrospective analysis	Adults (≥ 18 years) with signs and confirmation of dengue (N = 7)	Most patients with dengue appropriately received analgesic medication. The one patient administered treatment for severe malaria tested positive for malaria.
Oche, 2021 [27]	Nigeria	Cross-sectional	Health workers who had worked in a tertiary health center for at least one year (N = 367)	Overall, most health workers had adequate knowledge of dengue treatment. 89.7% of health workers believed that dengue in the presence of a comorbidity should be hospitalized. Most believed that intravenous fluid hydration and paracetamol should be administered to dengue patients (90.9% and 75.1%, respectively). Over one third of health workers believed that dengue patients should be administered antibiotics and antivirals (36.5%, 44.3%, respectively).
Sondo, 2018 [33]	Burkina Faso	Cross-sectional	Paramedical personnel (n = 224) and physicians (n = 32)	Both paramedical personnel and physicians had low levels of knowledge regarding dengue fever. In particular, paramedical personnels showed a weak knowledge of clinical forms and biological diagnosis. 37.5% of paramedical personnels and 3.1% of physicians believed that antibiotics should be prescribed to dengue patients. 48.7% of paramedical personnel and 3.1% of physicians believed that antimalarials should be prescribed to dengue patients.
Sondo, 2022 [32]	Burkina Faso	Cross-sectional	Hospitalized patients for suspected dengue fever (N = 1201)	Treatments administered to dengue patients include analgesics (100%) and anti-inflammatories (22%). While only 24.6% of patients had co-infection with malaria, 26.3% self-administered antimalarials and 92.6% were prescribed antimalarials by a healthcare worker before.
Sow, 2016 [9]	Senegal	Cross-sectional	13,845 patients, including 7387 with malaria and 41 with acute arbovirus infections (12 YFV, nine ZIKV, 16 CHIKV, three DENV, and one RVFV) were enrolled	This study showed that co-infections between Plasmodium spp. and arboviruses are frequent in Kedougou where competent vectors of both diseases are abundant. Vector competence and co-infection of certain malaria vectors, also regularly found infected by arboviruses, deserves further investigation. The frequent detection of arboviral disease outbreaks in the Kedougou region highlights the need to strengthen surveillance of AFI for a better estimation of human impact of arboviruses, as well as morbidity and mortality associated with concurrent malaria and arboviral infections. Finally, the high-grade fever ≥40 °C suggests the possibility of malaria and arboviral infection and should help to establish prompt and better care of individuals.

**Abbreviations:** AgNS1: NS1 Antigen; CSPS: health and social promotion center; IgG: Immunoglobulin G; IgM: Immunoglobulin M; PC: platelet concentrate; RDT: rapid diagnostic test

The majority of excluded studies (78%; 22 out of 28) did not report data on clinical management and were therefore excluded. In total, ten studies met the inclusion criteria [9,29–37]. Of these, three studies focused on healthcare workers [31,34,36], while six studies involved patients with dengue [9,29,31,32,34,38]. Geographically, seven studies were conducted in Burkina Faso [30,32–37], one in Ivory Coast [29], one in Senegal [9] and one in Nigeria [31].

### Diagnostic methods

Details regarding the types of diagnostic methods are summarized in **Table 3**. Serologic testing was conducted in all ten included studies, with most studies (8 out 10) assessing NS1 antigen, IgG, and IgM antibodies [29,30,32–37]. Two studies tested for IgM only [9,29]. Notably, in a study assessing healthcare workers’ knowledge of dengue in Burkina Faso, 29.5% (66/244) of paramedical personnel and 9.4% (3/32) of physicians reported being unable to interpret dengue serology results [36].

**Table 3:** Diagnostic methods used in studies.

Diagnostic test	Number of studies using test (% of total)
Serological testing – NS1 antigen, IgG and IgM antibodies	8 (80%)
Rapid diagnostic tests	5 (50%)
RT-PCR	4 (40%)
Serological testing – IgM only	2 (20%)

Abbreviations: IgG: immunoglobulin G; IgM: Immunoglobulin M; NS1: non-structural protein 1; RT-PCR: reverse transcription-polymerase chain reaction

Rapid diagnostic tests (RDTs) were used in five studies, most commonly the SD BIOLINE Dengue Duo (DENGUE NS1 Ag + IgG/IgM) test [30,32–35]. However, one study did not specify the type of serologic test used [37].

Molecular tests using dengue reverse transcription-polymerase chain reaction (RT-PCR) were performed alongside RDTs in four studies [9,29,32,33], although, RT-PCR was not consistently applied to all patients in withing these studies. In one study, a home-developed ELISA was used for all participants [29].

Several studies noted that arboviral infections, including dengue, were only considered by healthcare workers when malaria tests results were negative. The diagnostic hierarchy increases the risk of misdiagnosis and contributes to the under-reporting of concurrent infections [9].

### Disease management

Disease management strategies varied across the included studies and are summarized in **Table 4**. In general, physicians reported that the treatment pathways for dengue were predominantly guided by symptomatology rather than standardized protocols [34,36]. As such, dengue cases were generally treated with antipyretics, including analgesics and NSAIDs [29–31,33–35]. In several studies, dengue-positive patients were also treated with intravenous (IV) fluid transfusions [29,30,37]. For instance, in a 2018 study from Burkina Faso by Sondo et al. [36], 41% (106/256) of healthcare workers reported that they would administer transfusions to patients with dengue fever.

**Table 4:** Disease management strategies discussed in studies.

Management strategy	Number of studies discussing strategy (%)
Treatment with antipyretics	6 (60%)
Hydration	5 (50%)
Intravenous fluid transfusions	4 (40%)
Self-medication	4 (40%)
Dengue and other disease co-infection management	1 (10%)

Hydration was another common component of supportive care, discussed in five studies. In one study, 96% (94/98) of patients with dengue received rehydration therapy [30]. Among hospitalized patients, dialysis was used in 2.9% (10/346) of cases in Ouagadougou and 14% (1/7) in Abidjan [29,33]. Awareness of the importance of hydration was high among healthcare workers as 91% (310/357) in Nigeria and 41% (106/256) in Burkina Faso endorsed its use in dengue management [31,36].

Management strategies for dengue-malaria co-infections were only discussed in one study, where 90% (306/357) of healthcare professionals recommended hospitalization for patients with dengue and other co-existing diseases, although specific co-infections were not detailed [31]. In another study, 29% (4/14) of hospitalized patients who died from severe dengue and 25% (44/179) of patients with complicated clinical forms were also co-infected with malaria, though the specific clinical management strategy for these cases was not reported [35].

Self-medication practices were addressed in four studies [30,32,33,35]. In three studies, more than 30% of patients self-medicated: 18/58, 274/696, and 70/179, respectively [32,33,35]. One study reported that patients self-medicated with antimalarials (26%; 47/179) and analgesics (23%; 41/179) (32). Another study found that 71% (267/374) of patients self-treated with antimalarial drugs [33]. Traditional medicine use was also reported, with 4% (7/179) and 5.5% (14/261) of patients in Ouagadougou indicating such practices [31,33], and 3.6% (2/58) of participants in another study seeking care from traditional healers [30]. Sondo et al. [33] suggested that the widespread use of self-medication may have contributed to the observed rates of severe dengue and associated liver complications.

### Antibiotic misuse

Patient antibiotic use was described in two studies conducted in Burkina Faso and are summarized in **Table 5**. In a cross-sectional study across 15 public and private health centers, Sondo et al. [33] found that 25% (73/292) of patients with dengue received antibiotics. Similarly, Diallo et al. [30], in a retrospective analysis of patient records from a hospital in Ouagadougou, reported antibiotic use in 37% (36/98) of patients.

**Table 5:** Antibiotic misuse reported in studies.

Author name and year	Antibiotic prescription – total patients (%)
Sondo et al. 2017	73/292 (25%)
Diallo et al. 2017	36/98 (37%)

Three studies evaluated antibiotic prescribing practices among healthcare professionals treating patients with dengue [31,34,36]. Zongo et al. [34] conducted a qualitative study involving health professionals from six health and social promotion centers in Ouagadougou. The study found that antibiotics were often prescribed when dengue test results were negative, especially when a bacterial infection was suspected.

Oche et al. [31] in a study assessing knowledge, attitudes, and practices of health workers in Sokoto state, Nigeria, reported that 36.5% (123/357) of respondents believed antibiotics should be administered to patients with suspected dengue infection.

Similarly, the 2018 study by Sondo et al. [36], found that 3.1% (1/32) of physicians and 37.5% (84/224) of paramedical personnel supported prescribing and using antibiotics for patients with dengue.

### Antimalarial use

**Fig 2:**
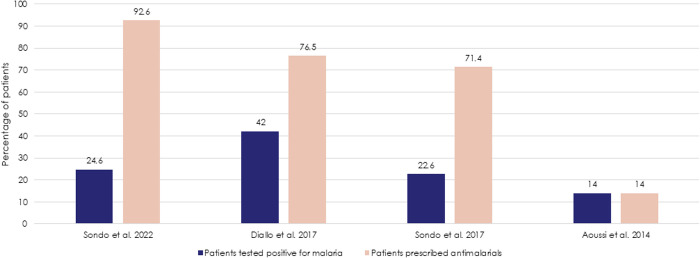
The percentage of patients prescribed antimalarials compared to patients positive for malaria (positive diagnostic test).

Antimalarials drugs were reportedly prescribed to patients in six studies conducted across West Africa [29,30,33–36]. In a retrospective analysis from a teaching hospital in Abidjan, antimalarial use was justified in seven suspected dengue cases, all of whom had positive malaria test results [29]. However, in three studies, the number of patients receiving antimalarials exceeded the number of those with confirmed malaria diagnoses. For instance, in the 2018 study by Sondo et al. [33], over 54% of patients were either prescribed or self-medicated with antimalarials drugs, despite only 23% (183/811) testing positive for malaria – raising concerns about the potential for antimalarial drug resistance. Similarly, Diallo et al. [30] reported that 66% (65/98) of patients received anti-malarial treatment, although only 43% (42/98) tested positive for malaria. In another study from Ouagadougou, 93% (166/179) of hospitalized patients with dengue were prescribed antimalarials, and 26% self-medicated with them, even though only 25% (44/179) had a confirmed dengue-malaria co-infection [35] (**Fig 2**).

In a study by Zongo et al. [34] healthcare practitioners in Burkina Faso acknowledged prescribing antimalarials despite negative dengue RDTs, citing concerns about false negative malaria test results. Similarly, Sondo et al. [33] reported that 43% (110/256) of healthcare workers – 49% [109/224] of paramedical personnel and 3.1% [1/32] of physicians – used antimalarials to treat dengue cases. According to Sondo and al [36], this practice was partially driven by the misconception among healthcare providers that dengue represents a complicated form of malaria, prompting antimalarial treatment even in the absence of confirmed malaria infection.

### Impact of differential diagnosis on treatment and management

Zongo et al. [34] was the only study that specifically evaluated the impact of dengue diagnosis on clinical management. Healthcare professionals recognized the value of dengue RDTs, as these tools facilitated differential diagnoses and improved dengue management. Clinical management was guided by both confirmed diagnosis and symptoms presentation. For patients diagnosed with dengue, healthcare workers commonly prescribed analgesics for pain relief, antipyretics to reduce fever, antibiotics when bacterial infections were suspected, and anti-emetics to manage vomiting. Notably, healthcare workers emphasized that they would avoid administrating anti-inflammatory medications in cases of confirmed positive dengue, due to the associated risk of hemorrhagic complications.

## Discussion

To our knowledge, this is the first literature review to synthesize existing data on current dengue diagnosis and management strategies specifically in West Africa. Previous reviews have largely focused on broader diagnostic algorithms and ideal management strategies without emphasizing the regional context of West Africa [39–41]. Accurate differential diagnosis is essential not only for timely and appropriate clinical care but also for informing surveillance systems, assessing the true burden of epidemics, and implementing effective public health control measures [42].

This review underscores the scarcity of literature on arboviral diseases in West Africa and highlights the crucial need to strengthen both diagnostic capacity and clinical management of dengue. Despite the endemic nature of dengue in the region, only ten studies meeting the inclusion criteria were identified. The widespread use of RDTs may be attributed to their affordability, ease of use, portability, and quick turnaround time. In contrast, molecular tests such as RT-PCR – which offer higher specificity and sensitivity – were less frequently employed, likely due to limited availability and infrastructure. These constraints impede the generation of reliable clinical data and hinder efforts to quantify the extent of misdiagnosis, a well-documented public health issue in the region [43,44].

Furthermore, molecular testing is recommended in regions where cross-reactivity with *Flaviviruses* is likely, such as West Africa according to the US Centers for Disease Control and Prevention (CDC) [45]. This is particularly important because individuals previously exposed to other *Flaviviruses* – such as Yellow fever, West Nile, and Zika virus – may produce false-positive results in serologic dengue diagnostic tests, complicating accurate diagnosis [9,46]. IgM antibody results, in particular, are prone to cross-reactivity, making interpretation difficult in flavivirus-endemic areas.

This review further highlights inappropriate clinical management practices reported in studies in Burkina Faso, Ivory Coast, Senegal, and Nigeria, were dengue cases were often treated with antibiotics, antimalarials, and anti-inflammatory drugs without confirmed indications. These findings reinforce the urgent need for routine, accurate differential diagnosis of non-malarial febrile illness using sensitive and specific diagnostic tools in healthcare settings. Inappropriate antibiotic use is of particular concern, given its contribution to the global rise of AMR, with Africa already experiencing the highest AMR-related mortality rates globally.

Evidence from other regions, including Colombia and India, has shown that the use of dengue RDTs is associated with a reduction in unnecessary antibiotic prescriptions [47,48]. Future research is needed to explore this association in West Africa, as only one study included in this review assessed the impact of dengue diagnosis on clinical management and decision-making. It is also important to recognize that while antibiotics are not indicated for viral infections, secondary bacterial infections can occur in the course of dengue illness. Clinicians should be equipped to identify these cases and apply appropriate diagnostic strategies to guide antibiotic use, thereby reducing morbidity, mortality, and resistance development.

Similar to antibiotic misuse, several studies in this review revealed that antimalarial drugs were frequently administered inappropriately. This aligns with existing evidence indicating that most febrile illnesses in sub-Saharan Africa are presumptively treated as malaria, regardless of diagnostic confirmation [19]. Such practices reflect the historical prioritization of malaria in global health programs, which may contribute to diagnostic results being overlooked in clinical decision-making [13]. Addressing this issue requires improved training of the health workforce, both through integration into medical curricula and ongoing professional development initiatives. These efforts would complement improved diagnostic capacity by equipping healthcare workers with the knowledge needed to interpret results appropriately and avoid unnecessary treatments. For instance, Oche et al. [31] found that fewer than half of healthcare workers (45%; 161/357) interviewed in Nigeria had received any training in the past year on managing hemorrhagic fevers, including dengue. Similarly, Zongo et al. [34] demonstrated that a targeted educational intervention on dengue diagnosis led to a shift in clinical practice away from routine antimalarial use. Sondo et al. [36] also emphasized the need for further training among healthcare workers in health and social promotion centers in Ouagadougou, which are often the first point of contact for patients. Many respondents in their study lacked sufficient knowledge about dengue prevention and management, further underlining the importance of education to ensure rational and evidence-based clinical care for febrile illnesses.

This review highlights several important gaps in the current literature. First, no eligible studies were identified on chikungunya, despite its recognition as a common cause of febrile illness in West Africa [49]. Second, many studies were excluded during the selection process due to the absence of information on clinical management, underscoring a broader deficiency in data related to the treatment and the care of dengue and chikungunya in the region. Third, very few studies evaluated the impact of dengue or chikungunya diagnosis on subsequent clinical management, limiting insight into how diagnostic confirmation – particularly RDTs – influences clinical decision-making. Fourth, co-infections with other pathogens (e.g., Zika, COVID-19, leptospirosis) present additional diagnostic challenges, yet these were addressed in only three included studies [35,50,51]. The underreporting of co-infections likely reflects the broader issue of limited diagnostic infrastructure and testing capacity for arboviruses in resource-limited settings [27]. The paucity of testing for other arboviruses is primarily attributable to limited laboratory capacity, lack of diagnostic infrastructure, and the absence of validated, affordable point-of-care tests (POCT) in low-resource settings. These factors collectively hinder comprehensive arboviral testing, leading to underdiagnosis of co-circulating viruses. Finally, most studies included in this review described clinical management practices in a narrative form, without systematically assessing the determinants or correlates of these practices. This represents a missed opportunity to identify actionable drivers of suboptimal management and to inform targeted interventions.

Several limitations should be considered when interpreting the findings of this review. First, the s methodological quality of the included studies was not assessed for internal or external validity. While this was beyond the scope of the review, which aimed to explore the breadth rather than the depth of existing literature, it limits the ability to draw definitive conclusions about the robustness of individual studies. Second, the small number of studies included may not be representative of the broader West African context. Many health centers in the region lack the infrastructure for diagnostic testing and rely primarily on clinical symptomatology for diagnosis [40], which may contribute to both underreporting and misclassification. Third, the limited volume of research on dengue diagnosis and management in West Africa underscores a pressing need for further investigation in this area. Dengue poses a significant economic burden; global estimates suggest an annual cost of approximately US$8.9 billion—surpassing that of other major tropical infectious diseases such as cholera and Chagas disease [52–54]. Moreover, the frequent misdiagnosis of malaria not only leads to inappropriate treatment but also incurs substantial costs in terms of patient morbidity and mortality due to misallocated healthcare resources. Finally, the incidence of dengue in West Africa is likely to increase due to a combination of population growth, climate change, rapid urbanization, poor sanitation infrastructure, and persistent poverty [13]. These contextual factors emphasize the urgent need for enhanced disease surveillance, improved vector control strategies, accurate differential diagnosis, and effective clinical management tailored to resource-constrained settings.

Several recommendations emerge from this literature review to improve dengue diagnosis and management in West Africa:

1. Integrate targeted training on arboviral diagnostic serology interpretation into medical education. Awareness campaigns or online modules focused on the interpretation of serological tests for arboviruses should be incorporated into medical school curricula and continuing professional development programs to enhance healthcare workers’ diagnostic capabilities.
2. Promote systematic education on the rational use of antibiotics and antimalarials. Medical training should include structured lessons on antimicrobial stewardship, particularly emphasizing appropriate prescription practices in the context of febrile illnesses and suspected co-infections. This is crucial to reduce the misuse of medications and mitigate the risk of antimicrobial resistance.
3. Emphasize the clinical relevance of co-infections in differential diagnosis. Clinical protocols and training should encourage the routine consideration of common co-infections (e.g., dengue and malaria) in febrile patients. This would support more accurate diagnoses and improve patient outcomes by guiding context-specific, evidence-based clinical management.

## Conclusion

There is a notable lack of comprehensive evidence on the diagnosis and management of dengue in West Africa, with most studies highlighting significant gaps in clinical practices, particularly the misuse of antibiotics and antimalarials. This review underscores the urgent need for improved diagnostic methods and better clinical management strategies for febrile illnesses in the region. The inappropriate use of antibiotics and antimalarials contributes to the rise of antimicrobial resistance, further complicating patient care.

Future research should focus on evaluating the impact of accurate, differential, and systematic diagnostic approaches on patient management, particularly in the context of co-infections. Studies should explore how the use of diagnostic tools, such as rapid tests and molecular diagnostics, influences treatment decisions. Furthermore, addressing healthcare worker training and improving awareness of the importance of proper diagnosis in febrile illness management are critical steps toward enhancing patient outcomes and reducing the burden of antibiotic resistance. Evidence-based strategies are essential for optimizing the clinical management of dengue, chikungunya, and malaria, ultimately improving healthcare delivery in West Africa.
